# Screening and treatment for tuberculosis in a cohort of unaccompanied minor refugees in Berlin, Germany

**DOI:** 10.1371/journal.pone.0216234

**Published:** 2019-05-21

**Authors:** Stephanie Thee, Renate Krüger, Horst von Bernuth, Christian Meisel, Uwe Kölsch, Valerie Kirchberger, Cornelia Feiterna-Sperling

**Affiliations:** 1 Department of Pediatrics, Division of Pneumonology and Immunology with Intensive Medicine, Charité Universitätsmedizin, Berlin, Germany; 2 Department of Immunology, Labor Berlin, Charité Vivantes GmbH, Berlin, Germany; 3 Berlin Center of Regenerative Therapies, Charité Universitätsmedizin, Berlin, Germany; Saint Louis University, UNITED STATES

## Abstract

**Introduction:**

In 2015, 4062 unaccompanied minor refugees were registered in Berlin, Germany. According to national policies, basic clinical examination and tuberculosis (TB) screening is a prerequisite to admission to permanent accommodation and schooling for every refugee. This article evaluates the use of an interferon-γ-release-assay (IGRA) during the initial examination and TB screening of 970 unaccompanied minor refugees.

**Results:**

IGRA test were obtained during TB screening for 301 (31.0%) of 970 adolescents not previously screened for TB. Positive IGRA results were obtained in 13.9% (42/301). Most of the 42 IGRA-positive refugees originated from Afghanistan or Syria (n?20 and 10 respectively). Two IGRA-positive adolescents were lost to follow-up, 2 were diagnosed with TB and the remaining 38 diagnosed with latent TB infection (LTBI). Demographic features of the 40 patients with positive IGRA result were as follows: 39 male, median age 16.8 years (IQR 16.0–17.2y), none meeting underweight criteria (median BMI 21.3kg/m^2^). On initial chest X-ray 2/40 participants had signs of active TB, while in 38 active disease was excluded and the diagnosis of latent TB infection (LTBI) made. Active hepatitis B-co-infection was diagnosed in 3/38 patients. All patients with LTBI received Isoniazid and Rifampicin for 3 months without occurrence of severe adverse events. The most frequently observed side effect was transient upper abdominal pain (n = 5). Asymptomatic elevation of liver transaminases was seen in 2 patients. 29 patients completed treatment with no signs of TB disease at the end of chemoprevention and 9 were lost to follow up.

**Conclusion:**

Screening for TB infection in minor refugees was feasible in our setting with a relatively high rate of TB infection detected. Chemopreventive treatment was tolerated well regardless of underlying hepatitis-B-status. Minor refugees migrating to Germany should be screened for TB infection, instead of TB disease only, regardless of the background TB incidence.

## Introduction

In 2015, around 1.1 million refugees were registered in Germany with estimated 40,000–60,000 unaccompanied minor refugees. Most of the unaccompanied minors originated from Afghanistan (34%), Syria (31%), Iraq (8.4%), Eritrea (8.1%) and Somalia (4.5%) [1]. In the city of Berlin, 4062 minor refugees were registered in 2015.

According to German national policies, every refugee has to undergo basic clinical examination and infectious tuberculosis (TB) is to be ruled out as soon as possible after entry to the country. This is a prerequisite to admission to permanent accommodation and schooling. Besides, vaccinations against tetanus, diphtheria, pertussis, poliomyelitis, mumps, measles and rubella are offered in case of incomplete or unknown immunization status.

TB screening is based on a chest X-ray in persons older than 15 years of age. In those younger than 15 years of age, making about 25% of all refugees, an interferon-γ-release-assay (IGRA) or the tuberculin skin test (TST) have been proposed for screening purposes [2] [3]. In case of symptoms of TB or a positive TST or IGRA a chest radiograph is obtained also in those being under 15 years of age.

A previous study informs about screening for infectious diseases except tuberculosis in minor refugees [4]. But regarding the burden of TB infection and TB disease, a substantial lack of knowledge exists for this cohort.

In many unaccompanied minors no TB screening or vaccinations were performed upon arrival to Germany, impeding schooling and further integration, because special initial acceptance facilities, so-called clearing offices, for unaccompanied minor refugees had to be established. Additionally, there was uncertainty on how to perform medical procedures in lack of a legal guardian.

In an action campaign in January 2016, initiated by the Charité—Universitätsmedizin Berlin (“Charité hilft”), unaccompanied refugees <18 years of age residing in Berlin were offered initial basic clinical examination, TB screening as well as the vaccinations mentioned above. For logistical reasons, an IGRA (QuantiFERON®-TB Gold In-Tube) was performed in those not having received TB screening (either TST, IGRA or chest-X-ray) before regardless of age.

This article provides information on the initial tuberculosis screening of unaccompanied minor refugees, focusing on those with positive IGRA results. Participants diagnosed with TB or latent tuberculosis infection (LTBI) received treatment according to national standard of care and were followed up prospectively. This data also adds to the limited body of evidence on chemopreventive TB treatment in adolescents, an age-group being considered as being more susceptible to drug induced hepatotoxicity compared to younger children [5, 6].

## Methods

Local authorities as well as several local charity organizations caring for unaccompanied minor refugees were contacted and informed about the screening campaign and asked to inform the refugees they care for. All minors participating received information on the screening and vaccination procedures in their mother tongue beforehand and were asked for written consent. Additionally, a questionnaire on the medical history including information on previous TB screening was asked to be filled in. Inclusion criteria were children and adolescents younger than 18 years of age with no signs of acute illness. In case of acute illness, the person was referred to a local medical service. At the day of the screening, interpreters of all languages mainly spoken by the refugees were available.

Participants were interrogated for any symptoms or complains and received a basic clinical examination. In case no TB screening had been performed, blood for the IGRA-test was taken by venipuncture. Vaccinations were offered if evidence of a complete immunization status was missing.

Blood samples for IGRA tests were transferred to the laboratory at site within two hours. Participants with a positive IGRA test (IFN-γ > 0.35 IU/mL) were followed up in the paediatric infectious disease clinic of the Charité University medicine. A chest X-ray was performed. In the absence of signs for active TB, the diagnosis of LTBI was made and chemopreventive treatment recommended. According to national guidelines, 3 months of isoniazid (INH) in combination with rifampicin (RMP) are recommended for chemopreventive therapy if no drug resistance was suspected. Because the index cases were not known in our cohort, susceptibility to INH and RMP was assumed and treatment started accordingly. In case of signs for TB disease, further diagnostic procedures according to national standards were performed and TB treatment initiated accordingly.

Before initiation of chemopreventive treatment, a baseline laboratory examination including whole blood cell count, liver transaminases, alkaline phosphatase, creatinine, uric acid and hepatitis B and C serology was performed. Patients were followed up clinically and laboratory tests were repeated after 2 and 6 weeks. At each follow-up visit, adherence was checked by either interrogation, pill count or inspection of urine for red-orange-coloring (due to RMP intake). Before chemoprevention was stopped and if possible one year after the end of chemoprevention, further chest X-rays were performed to exclude development of TB disease due to non-adherence, drug resistance or treatment failure.

## Results

Nine-hundred-seventy adolescents (median age 16.8 years, range 13.6–17.1years) participated in the screening campaign. Of 970 participants, 301 had not been screened for TB. The leading countries of origin of these participants were Afghanistan (125/301; 41.5%), Syria (69/301; 22.9%), Lebanon and Iran (both 14/301, 4.7%). 26 adolescents (8.6%) refused to indicate their country of origin. An IGRA-test was performed for TB screening, with 42 (13.9%) tested positive (see Table 1 for distribution according to country). Two of these adolescents were lost to follow-up, the remaining 40 were seen at the paediatric infectious disease clinic Charité University Medicine, Berlin.

**Table 1:** Country of origin of participants according to WHO world regions[37], number of participants receiving interferon-γ-release-assay (IGRA) -testing (n = 301) and numbers of positive (IGRA) test results (n = 42) according to country of origin.

*World region of origin*	*Number participants*	*IGRA test positive/ number tested per world region*	*Country of origin*	*IGRA test positive/ number tested per country*
*African Region*	59	3/23		
			Benin	1/1
			Chad	0/3
			Eritrea	0/2
			Gambia	2/8
			Ghana	0/1
			Guinea	0/2
			Guinea-Buissau	0/1
			Kenya	0/2
			Mali	0/2
			Somalia	0/1
*South-East Asia Region*	12	2/7		
			Bangladesh	1/4
			India	1/2
			Vietnam	0/1
*Eastern Mediterranean Region*	825	32/237		
			Afghanistan	19/125
			Iran	0/14
			Iraq	1/5
			Kurdistan	0/1
			Lebanon	1/14
			Libya	1/1
			Morocco	1/2
			Pakistan	0/5
			Syria	9/69
			Tunisia	0/1
*European Region*	17	1/8		
			Albania	0/1
			Moldova	0/1
			Palestine	0/4
			Turkey	0/1
			Turkmenistan	1/1
*Unknown*	57	4/26		4/26
**Total**	**970**	**42/301**		**42/301**

Demographic features of this cohort were as follows: 39 male, 1 female, median age 16.8 years (IQR 16.0–17.2y), median weight 62.5kg (IQR 58.4–66.5), median BMI 21.3kg/m^2^ (IQR 20.3–22.6kg/m^2^). Therefore, none of the participants met underweight criteria [7]. Positive IGRA-test results varied from 0.39–15.46 IU/ml (median 3.39IU/ml). In the adolescent with an IGRA-result of 0.39IU/ml a repeated test one month later showed a result of 1.26IU/ml.

The initial chest X-ray showed signs of intrathoracic TB in 2 cases (one from Syria, one from Afghanistan) being transferred to a paediatric clinic for further diagnostic work-up and treatment. One of these adolescents reported weight loss and fatigue, but both did not have symptoms like persistent cough or night sweats. One patient had hilar lymphnode TB and the other pulmonary TB, both without detection of M. tuberculosis in sputum. In another adolescent with positive IGRA-test, a small pleural effusion was suspected that cleared without treatment. A further patient reported of cough for more than 8 days and night sweats without weight loss, but had a normal chest X-ray.

In 38 adolescents, the diagnosis of LTBI was made and chemoprevention with 3 months INH/RMP recommended. Four patients were seen at the initial visit only. Of 29 patients seen at 3 months before stopping treatment, 10 had been seen for follow up visits while being on chemopreventive therapy once and 19 twice, respectively. Four patients were seen during treatment, but lost to follow up at the 12 weeks visit. Main reason for lost to follow up was relocation to another city. At the three months visit, a chest X-ray was taken again before chemoprevention was stopped. Twenty-eight of 29 chest X-rays were reported to be normal. Nine patients received a chest x-ray one year after completion of chemoprevention and none developed TB. In one patient, very small (<1 cm) opacifications with calcifications in the right upper lobe were detected in the follow-up chest X-rays that had been missed in the initial report. In this patient, the diagnosis of previous TB was made retrospectively.

Testing for hepatitis B and C was performed in 38/38 cases, and active hepatitis B infection was diagnosed in 3 cases. Initial viral load was 178, 105 and >70 000 000 HBV-DNA IE/ml, respectively. There was no case of hepatitis C. Baseline liver transaminases were slightly elevated (< 3times upper limit) in 3 cases of which 1 had active hepatitis B. This patient had a viral load >70.000,000IE/ml and was started on treatment with entecavir at the end of chemopreventive TB treatment. Anaemia was found at baseline in 3 patients (minimal Hb 9.0g/dl); no further abnormalities were noted.

Non-adherence was suspected in 10 adolescents during treatment followed by counselling again. At the end of treatment non-adherence was only suspected in 1/29 patients. No severe adverse events occurred and treatment had not to be stopped due to side effects in any case. Transient upper abdominal pain was reported in 5 cases. One patient with chronic hepatitis B had permanent gastric discomfort. One patient complained about alopecia. Asymptomatic elevation of liver transaminases above 3 times upper normal limit (but <5times upper normal limit) occurred in two patients (one chronic hepatitis B co-infection). Patients were monitored for signs of increased hepatotoxicity, but therapy could be continued until completion of 3 months therapy. Transaminases normalized or decreased with cessation of treatment with INH and RMP.

## Discussion

Concordant with official German refugee reports, the majority of our cohort originated from Afghanistan and Syria. According to WHO country reports, Afghanistan has a TB incidence of 189/100.000, while Syria is considered a low TB burden country with an estimated incidence of 19/100.000 [8]. For comparison, TB incidence was 7.2/100.000 in Germany in 2016 [9].

Two patients of our cohort with a positive IGRA (n = 42) had radiographic signs of active TB. Because persons with acute illness were excluded from our campaign, the true burden of TB in refugee minors might have been underestimated. Comparison with other studies is challenging because of the large diversity of policies on TB and TB infection screening in European countries [10]. In some European countries (e.g. Netherlands, Sweden, Spain) TB screening is restricted to asylum seekers originating from countries with a TB-incidence of more than 40-100/100 000, while others aim to screen all asylum seekers (e.g. Belgium, Germany) regardless of TB incidence in home countries according to WHO estimates [10]. In Switzerland, symptom triage screening is performed and only those classified as being at risk for TB are screened further using a chest X-ray (www.tb-screen.ch). Besides the targeted population, the target of TB screening itself varies from country to country. Sometimes, a TST or an IGRA is performed to detect tuberculosis infection (mainly in children or younger adults) while other focus on tuberculosis disease and primarily only a chest x-ray is taken.

In a study on a large cohort (n = 2936) of unaccompanied minors seeking asylum in Sweden in 2015, Bennet et al. reported numbers of TB disease of 500-3400/100,000 [11]. According to Swedish policy, screening is restricted to migrants from countries with a TB burden of more than 100/100,000 or if the screening doctor found other risk factors for TB. Therefore, 82% of the Swedish cohort originated from high burden countries (HBC), while in our study as well as in a further German pediatric study, migrants from HBC, besides Afghanistan, only form a small minority [12].

These higher numbers of TB found in the study by Bennet et al. as well as in our study are likely attributable to a high risk for both, TB infection and progression to disease, under conditions of conflict or humanitarian crisis in the country of origin as well as during transit to Europe [13]. Depending on many factors like background TB incidence, access to health care, nutrition, overcrowding, transit time, migration route and others, estimates of TB incidence can often dramatically exceed TB incidence of the country of origin [13] [14]. Transit time was asked for in all refugee minors of our cohort, but responds were often very imprecise or refused.

In our study, both adolescents with TB disease would have been missed in a symptom based approach. In a TB screening exclusively performed in refugees from HBC, only the patient from Afghanistan, but not the one from Syria would have been detected. In Finnish screening guidelines, the increased risk for TB in migrants from conflict areas has been taken into account and all migrants from Syria and Iraq are screened with chest X-ray while otherwise screening is restricted to countries with a TB incidence of more than 40/100 000 [10].

All patients in our study diagnosed with LTBI received a combination therapy with INH and RMP for 3 months. The medication was tolerated fairly well, no major side effects were observed. Hepatotoxicity is the most serious adverse effect related to INH with rare reports on acute liver failure even during chemopreventive therapy [15]. In previous studies on children treated with INH for LTBI, asymptomatic elevated liver enzymes were found in 5–10%, with a higher incidence in adolescents compared to adults [5, 16–20]. Besides INH-dose, disease severity, slow-acetylator status and co-medication with RMP have been identified as risk factors for hepatotoxicity [16]. Based on our experience in a German pediatric cohort, chemopreventive therapy with INH and RMP is very well tolerated [21].

The impact of chronic hepatitis B infection on antituberculotic drug-induced liver injury is discussed controversially and has been inconsistently identified as a risk factor for hepatotoxicity [22, 23]. Gray et al. identified abnormal baseline liver function tests to be associated with INH-associated hepatotoxicity in chemopreventive therapy, but no information on the underlying cause of elevated liver enzymes is provided [24]. Increase of liver transaminases above 3 times above the normal upper range was noted in two patients, one being a patient with active hepatitis B.

The adolescents of our study were accommodated in Berlin only temporarily, facing relocation at any time. These circumstances are not only reflected in the relatively high number of persons (n = 9/38) lost to follow-up at the end of chemopreventive treatment but also in the number of adolescents in whom non-adherence during chemopreventive therapy (n = 10) was suspected. However, following counselling, non-adherence at the end of chemoprevention was only suspected in one patient.

Treating LTBI is a cornerstone for elimination of TB in low incidence countries and growing evidence on tolerability and efficacy of chemopreventive treatment exists [25]. Numerous studies have shown that a majority of incident TB cases among migrants arise through the reactivation of LTBI probably acquired outside the host country. Epidemiological evidence also shows that progression from LTBI to tuberculosis disease usually occurs within the first two years after arrival [26, 27]. In a recent publication, results of 17 years LTBI screening in England among non-UK born individuals were evaluated, demonstrating a strong association between no chemoprophylaxis and developing TB disease [28]. Therefore, screening programs only aiming to identify adults with active TB neglect addressing the reservoir of tuberculosis and the risk of transmission of TB within refugee centers and to the resident population [29]. Concerns have arisen about the feasibility of LTBI screening in all pediatric refugees. While screening for TB infection was feasible in our setting, some patients were lost to follow up during chemopreventive treatment. In Germany, it is standard of care to perform a chest X-ray after completion of chemopreventive treatment. The risk of TB disease following treatment with INH and RMP has been reported to be approximately 4% and equal to treatment with INH monotherapy [30]. If a clinical follow-up or repetition of chest X-rays is the most appropriate way to monitor patient for the development of active TB is beyond the scope of the current study. Recently, data is emerging showing equal efficacy and safety of 3-4months RMP alone versus 6–9 months of INH with better adherence in the shorter RMP regimen [31]. With this data in mind, adding INH for chemoprevention might add to the risk for hepatoxicity without improving efficacy and has come under scrutiny. Nevertheless, there is no clear evidence demonstrating increased hepatotoxicity rates in children receiving combination therapy. At the time of the study, combination of INH and RMP has been standard therapy in Germany and endorsed by WHO LTBI treatment guidelines [32]

Any screening program faces screening in a high number of migrants to detect those who actually are infected with tuberculosis or have the disease [33]. For LTBI screening, the body of evidence for implementation, impact and cost-effectiveness as predicted in mathematical models is still limited and setting-specific [34, 35]. However, evidence exists for cost-effectiveness of school-based tuberculosis screening programs for children migrating to low-incidence countries [36]. For the greatest individual and public health benefits, more emphasis must be placed on strategies not only to ensure screening but also to facilitate treatment completion.

In summary, we report on a cohort of minor refugees originating from high but also from low TB burden countries with a high incidence of TB and LTBI. Screening for TB infection instead of TB disease was feasible and chemopreventive treatment with 3 months of INH and RMP tolerated well regardless of underlying hepatitis B status. Minor refugees migrating to Germany should be screened for TB infection, instead of TB disease only regardless of the background TB incidence. For the greatest individual and public health benefits, more emphasis must be placed on strategies not only to ensure screening but also to facilitate treatment completion.

## Supporting information

S1 File Questionnaire TB screening. (PDF)

S1 Dataset Minimal data set. (XLSX)
