# Supplementary material for: Screening and treatment for tuberculosis in a cohort of unaccompanied minor refugees in Berlin, Germany
Source: PLoS One. 2019 May 21;14(5):e0216234. doi: 10.1371/journal.pone.0216234 (PMC6528979; doi:10.1371/journal.pone.0216234)
Supplement: S1 File — (PDF) [file pone.0216234.s001.pdf]

CHARITÉ UNIVERSITÄTSMEDIZIN BERLIN	Dokumentation zur ärztlichen Erstaufnahmeuntersuchung von Asylsuchenden entsprechend §62 AsylG und §36IfSG in Berlin	fortlaufende Nr. *****

Stammdaten

Name: ______ Vorname: ______
Geb. Datum: ______ männl ☐ weibl ☐ Reg.-ID-Nr: ______
Ort der Erstaufnahmeuntersuchung: ☐Kruppstraße ☐Bundesallee 171 ☐ ______
Datum: ___ | ___ 20___ Uhrzeit: ___ : ___ Herkunftsland: ______
Sprache: ______ Beginn der Flucht: ___ | ___ 20___ Einreise nach Deutschland: ___ | ___ 20___
Anschrift Unterkunft: ______ Ansprechpartner /Betreuer: ______ Tel. ______
Bereits durchgeführt am: ______ ☐ Erstuntersuchung ☐ TBC-Screening ☐ Impfung

Allgemeine Anamnese

Vorerkrankungen

☐ Diabetes mell.
☐ Typ 1 | ☐ Typ 2
☐ Nieren
☐ Dialysepfl.
☐ ______
☐ Augenerkrank.
☐ ______
☐ Magen/Darm
☐ Diarrhoen
☐ Hauterkrank
☐ ______
☐ Herz-/Kreislauferkrankung
☐ KHK | Angina Pectoris
☐ Rhythmusstörung
☐ Klappendysfunktion
☐ Insuffizienz
☐ Bluthochdruck
☐ ______
☐ Atemwegserkrank.
☐ COPD | Asthma
☐ Pneumonie
☐ ______
☐ Rheuma-/Autoimmun Erkrankungen
☐ ______
☐ Infektionserkrank.
☐ TBC
☐ Hepatitis A | B | C
☐ HIV | AIDS
☐ ______
☐ OP´s:
☐ ______

Schwangerschaft:
Nicht bekannt ☐
Bekannt ☐
SSW: ______

Letzte Mensis: ______

Vitalparameter
*optional
Temp: ______ °C:
Gewicht: ______ Kg
Größe: ______ cm
P*: ______ /min
RR*: ______ mmHg

Dauer-/Medikamente

Name/Wirkstoff	Dosierung	Applikation
		☐ oral | ☐ supp | ☐ i.m. | ☐ s.c. | ☐ transdermal
		☐ oral | ☐ supp | ☐ i.m. | ☐ s.c. | ☐ transdermal
		☐ oral | ☐ supp | ☐ i.m. | ☐ s.c. | ☐ transdermal

Allergien
Nicht bekannt ☐
☐ ______
☐ ______
☐ ______

Symptome der letzten 3 Monate

Symptome

	Ja	Beginn	Nein		Ja	Beginn	Nein
**Husten**	☐		☐	**Durchfall**	☐		☐
**Auswurf**	☐		☐	**Erbrechen/Übelkeit**	☐		☐
Eitrig	☐		☐	**Blut/Schleim im Stuhl**	☐		☐
Blutig	☐		☐	**Juckreiz**	☐		☐
**Gewichts-verlust**	☐		☐	**Allg. Schwäche/ Müdigkeit**	☐		☐
**Nachtschweiß**	☐		☐	**Knöchelödeme**	☐		☐
**Auffällig. Unruhe**	☐		☐	**Schlaf-störungen**	☐		☐
**Blut im Urin**	☐		☐	**Hautausschlag**	☐		☐
**Fieber**	☐		☐	**Appetitlosigkeit**	☐		☐

Kürzel Arzt: ______

Körperliche Untersuchung

	Ja		nein
V.a. **Masern**	☐		☐
V.a. **Windpocken**	☐		☐
V.a. **Scabies**	☐	**Behandlung begonnen am:** ______	☐
V.a. **Kopfläuse**	☐	**Behandlung begonnen am:** ______	☐
**BCG Impfnarbe**	☐		☐
Anhalt f. **weitere Erkrankung**	☐ ______		☐

Kürzel Arzt: ______

Impfen

Impfstatus
Impfpass vorhanden ☐ ja | ☐ nein
Impfstatus vorhandener Impfungen
☐ Mumps ☐ Masern ☐ Röteln ☐ Tetanus ☐ Diphterie ☐ Pertussis ☐ Meningokokken C
☐ Polio ☐ Varizellen ☐ Hepatitis B ☐ BCG ☐ Pocken ☐ ______

Impfangebot nötig – falls ja erfolgen diese im Rahmen der Erstuntersuchung
☐ Mumps ☐ Masern ☐ Röteln ☐ Tetanus ☐ Diphterie ☐ Pertussis ☐ Meningokokken C
☐ Polio ☐ Varizellen ☐ Hepatitis B ☐ Andere, welche: ______

Kürzel Arzt: ______

TBC

TB-Spezifische Diagnostik

Diagnostik	Veranlasst	Datum	
Röntgen-Thorax (in einer Ebene)	☐		Ggf Kontroll-Röngten in ☐ 3 Monaten ☐ 6 Monaten
IGRA - Bluttest	☐		Kürzel Arzt: ______

Aus Sicht des Infektionsschutzes und der verpflichtenden Untersuchung nach §62AsylG, besteht aus medizinischer Sicht **kein** Bedenken gegen die Unterbringung in einer Gemeinschaftseinrichtung: ☐ Ja ☐ Nein ☐ **Untersuchung wurde verweigert**
Eine Impfberatung wurde durchgeführt: ☐ Ja ☐ Nein
Eine weitere Vorstellung ggf. Diagnostik wird empfohlen ☐ Ja ☐ Nein

Unterschrift/Stempel Arzt______

CHARITÉ UNIVERSITÄTSMEDIZIN BERLIN	Dokumentation zur ärztlichen Erstaufnahmeuntersuchung von Asylsuchenden entsprechend §62 AsylG und §36IfSG in Berlin	fortlaufende Nr. *****

Stammdaten

Name: ________________ Vorname: ________________

Geb. Datum: __________ männl ☐ weibl ☐ Reg.-ID-Nr: ________________

Ort der Erstaufnahmeuntersuchung: ☐Kruppstraße ☐Bundesallee 171 ☐ ________________

Datum: _____ | _____ 20_____ Uhrzeit: _____ : _____ Herkunftsland: ________________

Sprache: ________________ Beginn der Flucht: _____ | _____ 20_____ Einreise nach Deutschland: _____ | _____ 20_____

Anschrift Unterkunft: ________________ Ansprechpartner /Betreuer: ________________ Tel. ________________

Bereits durchgeführt am: ________________ ☐ Erstuntersuchung ☐ TBC-Screening ☐ Impfung

Allgemeine Anamnese

Vorerkrankungen

☐ Diabetes mell.
- ☐ Typ 1 | ☐ Typ 2

☐ Nieren
- ☐ Dialysepfl.
- ☐ ____________

☐ Augenerkrank.
- ☐ ____________

☐ Magen/Darm
- ☐ Diarrhoen

☐ Hauterkrank
- ☐ ____________

☐ Herz-/Kreislauferkrankung
- ☐ KHK | Angina Pectoris
- ☐ Rhythmusstörung
- ☐ Klappendysfunktion
- ☐ Insuffizienz
- ☐ Bluthochdruck
- ☐ ____________

☐ Atemwegserkrank.
- ☐ COPD | Asthma
- ☐ Pneumonie
- ☐ ____________

☐ Infektionserkrank.
- ☐ TBC
- ☐ Hepatitis A | B | C
- ☐ HIV | AIDS
- ☐ ____________

☐ Rheuma-/Autoimmun Erkrankungen
- ☐ ____________

☐ OP´s:
- ☐ ____________

Schwangerschaft:
Nicht bekannt ☐
Bekannt ☐
SSW: ____________

Letzte Mensis: ____________

Vitalparameter
*optional
Temp: ____________ °C:
Gewicht: ____________ Kg
Größe: ____________ cm
P*: ____________ /min
RR*: ____________ mmHg

Dauer-/Medikamente

Name/Wirkstoff	Dosierung	Applikation
		☐ oral | ☐ supp | ☐ i.m. | ☐ s.c. | ☐ transdermal
		☐ oral | ☐ supp | ☐ i.m. | ☐ s.c. | ☐ transdermal
		☐ oral | ☐ supp | ☐ i.m. | ☐ s.c. | ☐ transdermal

Allergien
Nicht bekannt ☐
☐ ________________
☐ ________________
☐ ________________

Symptome der letzten 3 Monate

Symptome

	Ja	Beginn	Nein		Ja	Beginn	Nein
**Husten**	☐		☐	**Durchfall**	☐		☐
**Auswurf**	☐		☐	**Erbrechen/Übelkeit**	☐		☐
Eitrig	☐		☐	**Blut/Schleim im Stuhl**	☐		☐
Blutig	☐		☐	**Juckreiz**	☐		☐
**Gewichts-verlust**	☐		☐	**Allg. Schwäche/ Müdigkeit**	☐		☐
**Nachtschweiß**	☐		☐	**Knöchelödeme**	☐		☐
**Auffällig. Unruhe**	☐		☐	**Schlaf-störungen**	☐		☐
**Blut im Urin**	☐		☐	**Hautausschlag**	☐		☐
**Fieber**	☐		☐	**Appetitlosigkeit**	☐		☐

Kürzel Arzt: ____________

Körperliche Untersuchung

	Ja		nein
V.a. **Masern**	☐		☐
V.a. **Windpocken**	☐		☐
V.a. **Scabies**	☐	**Behandlung begonnen am:** ____________	☐
V.a. **Kopfläuse**	☐	**Behandlung begonnen am:** ____________	☐
**BCG Impfnarbe**	☐		☐
Anhalt f. **weitere Erkrankung**	☐ ________________		☐

Kürzel Arzt: ____________

Impfen

Impfstatus
Impfpass vorhanden ☐ ja | ☐ nein
Impfstatus vorhandener Impfungen
☐ Mumps ☐ Masern ☐ Röteln ☐ Tetanus ☐ Diphterie ☐ Pertussis ☐ Meningokokken C
☐ Polio ☐ Varizellen ☐ Hepatitis B ☐ BCG ☐ Pocken ☐ ____________

Impfangebot nötig – falls ja erfolgen diese im Rahmen der Erstuntersuchung
☐ Mumps ☐ Masern ☐ Röteln ☐ Tetanus ☐ Diphterie ☐ Pertussis ☐ Meningokokken C
☐ Polio ☐ Varizellen ☐ Hepatitis B ☐ Andere, welche: ________________________

Kürzel Arzt: ____________

TBC

TB-Spezifische Diagnostik

Diagnostik	Veranlasst	Datum
Röntgen-Thorax (in einer Ebene)	☐	
IGRA - Bluttest	☐	

Ggf Kontroll-Röngten in ☐ 3 Monaten ☐ 6 Monaten

Kürzel Arzt: ____________

Aus Sicht des Infektionsschutzes und der verpflichtenden Untersuchung nach §62AsylG, besteht aus medizinischer Sicht **kein** Bedenken gegen die Unterbringung in einer Gemeinschaftseinrichtung: ☐ Ja ☐ Nein ☐ **Untersuchung wurde verweigert**

Eine Impfberatung wurde durchgeführt: ☐ Ja ☐ Nein

Eine weitere Vorstellung ggf. Diagnostik wird empfohlen ☐ Ja ☐ Nein

Unterschrift/Stempel Arzt________________

CHARITÉ UNIVERSITÄTSMEDIZIN BERLIN	Dokumentation zur ärztlichen Erstaufnahmeuntersuchung von Asylsuchenden entsprechend §62 AsylG und §36IfSG in Berlin	fortlaufende Nr. *****

Name: ______________________ Vorname: ______________________

Geb. Datum: __________ männl ☐ weibl ☐ Reg.-ID-Nr: ______________________

Ort der Erstaufnahmeuntersuchung: ☐Kruppstraße ☐Bundesallee 171 ☐______________

Datum: _____ | _____ 20____ Uhrzeit: _____ : _____ Herkunftsland: ______________

Sprache: ______________ Beginn der Flucht: _____ | _____ 20____ Einreise nach Deutschland: _____ | _____ 20____

Anschrift Unterkunft: ______________ Ansprechpartner /Betreuer: ______________ Tel. ______________

Bereits durchgeführt am: __________ ☐ Erstuntersuchung ☐ TBC-Screening ☐ Impfung

BESCHEINIGUNG

über die ärztliche Untersuchung entsprechend § 62 AsylG und § 36 IfSG.

Oben Genannter wurde entsprechend §62 des AsylG und §36 IfSG ärztlich

untersucht und hat eine Impfberatung erhalten.

Aus Sicht des Infektionsschutzes und der verpflichtenden Untersuchung nach §62AsylG, besteht aus medizinischer Sicht **kein** Bedenken gegen die Unterbringung in einer Gemeinschaftseinrichtung: ☐ Ja ☐ Nein ☐ **Untersuchung wurde verweigert**

Eine Impfberatung wurde durchgeführt: ☐ Ja ☐ Nein

Eine weitere Vorstellung ggf. Diagnostik wird empfohlen ☐ Ja ☐ Nein

Unterschrift/Stempel Arzt______________________

Aufklärung **Nr. 28: Tdap-IPV** **Englisch/English**

Name *Name* ____

Vaccination against Tetanus, Diphtheria, Whooping cough, Polio
Schutzimpfung gegen Wundstarrkrampf, Diphtherie, Keuchhusten und Kinderlähmung

Please find enclosed an information sheet on the vaccination procedure against tetanus, diphtheria, whooping cough, and polio (Tdap-IPV). It contains essential information about diseases preventable by vaccination, the vaccine, the vaccination, and immunization reactions and potential vaccine complications.
Anliegend erhalten Sie ein Merkblatt über die Durchführung der Schutzimpfung gegen Wundstarrkrampf, Diphtherie, Keuchhusten und Kinderlähmung (Td-ap-IPV). Darin sind die wesentlichen Angaben über die durch die Impfung vermeidbaren Krankheiten, den Impfstoff, die Impfung sowie über Impfreaktionen und mögliche Impfkomplikationen enthalten.

Before the vaccination is performed, the following additional information is requested:
Vor Durchführung der Impfung wird zusätzlich um folgende Angaben gebeten:

1. Is the vaccinee currently healthy?
   Ist der Impfling gegenwärtig gesund?

□ yes *(Ja)* □ no *(Nein)*

2. Has vaccinee experienced a disease of the nervous system, or does a seizure disorder exist?
   Hat der Impfling eine Erkrankung des Nervensystems durchgemacht oder besteht ein Krampfleiden?

□ yes *(Ja)* □ no *(Nein)*

3. Does the vaccinee have known allergies?
   Ist bei dem Impfling eine Allergie bekannt?

□ yes *(Ja)* □ no *(Nein)*

if so, which ones *wenn ja, welche?* ____

4. Did the vaccinee experience allergic reactions, high fever, or other unusual reactions after a previous vaccination?
   Traten bei dem Impfling nach einer früheren Impfung allergische Erscheinungen, hohes Fieber oder andere ungewöhnliche Reaktionen auf?

□ yes *(Ja)* □ no *(Nein)*

If you would like to know more about the vaccination against tetanus, diphtheria, whooping cough, and polio, ask your vaccinating doctor!
Falls Sie noch mehr über die Schutzimpfung gegen Wundstarrkrampf, Diphtherie, Keuchhusten und Kinderlähmung wissen wollen, fragen Sie den Impfarzt!

Please bring your vaccination passport to the vaccination appointment!
Zum Impftermin bringen Sie bitte das Impfbuch mit!

Aufklärung **Nr. 28: Tdap-IPV** **Englisch/English**

Consent

Einverständniserklärung

to vaccination against tetanus, diphtheria, whooping cough, and polio

zur Durchführung der Schutzimpfung gegen Wundstarrkrampf, Diphtherie, Keuchhusten und Kinderlähmung

(Carbon copy forms are also available for vaccinees or their guardians to receive a copy in accordance with patient's right law)

(Es stehen auch Formulare mit Durchschlag zur Verfügung, um den Impflingen bzw. ihren Sorgeberechtigten gemäß Patientenrechtegesetz eine Kopie mitgeben zu können.)

Name of vaccinee ____

Name des Impflings

Date of birth ____

geb. am

I have read the information in this leaflet my doctor explained in detail the information about the vaccination in a meeting.

Ich habe den Inhalt des Merkblatts zur Kenntnis genommen und bin von meinem Arzt/meiner Ärztin im Gespräch ausführlich über die Impfung aufgeklärt worden.

□ I have no further questions.
Ich habe keine weiteren Fragen.

□ I agree to the suggested vaccination against tetanus, diphtheria, whooping cough, and polio.
Ich willige in die vorgeschlagene Impfung gegen Wundstarrkrampf, Diphtherie, Keuchhusten und Kinderlähmung ein.

□ I reject the vaccination. I was informed about possible disadvantages of rejecting this vaccination.
Ich lehne die Impfung ab. Über mögliche Nachteile der Ablehnung dieser Impfung wurde ich informiert.

Comments: *Vermerke*____

Place and date: *Ort, Datum*____

Signature of vaccinee or legal guardian	Signature of doctor
Unterschrift des Impflings bzw. des Sorgeberechtigten	*Unterschrift des Arztes/der Ärztin*

Name____
Name

Vaccination against measles, mumps and rubella
Schutzimpfung gegen Masern, Mumps und Röteln

Please find enclosed an information leaflet about vaccination against measles, mumps and rubella. It contains essential information about illnesses that can be prevented by the vaccination, the vaccine, the vaccination, tips for after the vaccination, and also describes common reactions to the vaccination and possible complications.
Anliegend erhalten Sie ein Merkblatt über die Durchführung der Schutzimpfung gegen Masern, Mumps und Röteln. Darin sind die wesentlichen Angaben über die durch die Impfung vermeidbaren Krankheiten, den Impfstoff, die Impfung, das Verhalten nach der Impfung sowie über Impfreaktionen und mögliche Impfkomplikationen enthalten

Before the vaccination is administered, we request the following additional information:
Vor Durchführung der Impfung wird zusätzlich um folgende Angaben gebeten:

1. Does the person to be vaccinated have an immunodeficiency (congenital, acquired or caused by medication)?
Besteht bei dem Impfling eine Immunmangelkrankheit (erworben, angeboren, durch Medikamente bedingt)?

☐ Yes *(Ja)* ☐ No *(Nein)*

2. Did the person to be vaccinated receive immunoglobulin (gamma globulin) or a blood transfusion in the last 3 months?
Hat der Impfling in den vergangenen drei Monaten Immunglobulin (Gammaglobulin) erhalten oder wurde eine Bluttransfusion vorgenommen?

☐ Yes *(Ja)* ☐ No *(Nein)*

3. Has the person to be vaccinated received another vaccination in the last four weeks, or is there a vaccination against other illnesses planned in the next four weeks?
Wurde bei dem Impfling in den vergangenen vier Wochen eine Schutzimpfung durchgeführt oder ist in den kommenden vier Wochen eine Schutzimpfung gegen andere Erkrankungen geplant?

☐ Yes *(Ja)* ☐ No *(Nein)*

If yes, which ones and when: ________________________________
wenn ja, welche und wann?

4. For vaccinations of women of child-bearing age: are you currently pregnant?
Bei Impfung von Frauen im gebärfähigen Alter: Besteht zurzeit eine Schwangerschaft?

☐ Yes *(Ja)* ☐ No *(Nein)*

If you would like to know more about the vaccination against measles, mumps and rubella, please ask the vaccinating physician.
Falls Sie noch mehr über die Schutzimpfung gegen Masern, Mumps und Röteln wissen wollen, fragen Sie den Impfarzt!

Please bring your vaccination record to your vaccination appointment.
Zum Impftermin bringen Sie bitte das Impfbuch mit!

Declaration of consent
Einverständniserklärung

to vaccinate the person listed below against measles, mumps and rubella
zur Durchführung der Schutzimpfung gegen Masern, Mumps und Röteln

Name of person to be vaccinated: ____
Name des Impflings

Date of birth: ____
geb. am

I have read the information leaflet and have received detailed information about the vaccination during the consultation with my physician.
Ich habe den Inhalt des Merkblatts zur Kenntnis genommen und bin von meinem Arzt/meiner Ärztin im Gespräch ausführlich über die Impfung aufgeklärt worden.

☐ I have no further questions.
Ich habe keine weiteren Fragen.

☐ I give my consent to the recommended vaccination against measles, mumps and rubella.
Ich willige in die vorgeschlagene Impfung gegen Masern, Mumps und Röteln ein.

☐ I decline the vaccination. I have been informed about the possible disadvantages of declining this vaccination.
Ich lehne die Impfung ab. Über mögliche Nachteile der Ablehnung dieser Impfung wurde ich informiert.

Notes: *Vermerke*____

Place and date: *Ort, Datum* ____

_______________________________ _______________________________

Signature of person to be vaccinated or his/her legal guardian<br>*Unterschrift des Impflings bzw. des Sorgeberechtigten*	Doctor’s signature<br>*Unterschrift des Arztes/der Ärztin*

Name____
Name

Vaccination against chickenpox
Schutzimpfung gegen Windpocken

Please find enclosed an information leaflet about the vaccination against chickenpox. It contains essential information about illnesses that can be prevented through the vaccination, the vaccine used and the vaccination itself. It also describes reactions to the vaccination and possible complications.
Anliegend erhalten Sie ein Merkblatt über die Durchführung der Schutzimpfung gegen Windpocken. Darin sind die wesentlichen Angaben über die durch die Impfung vermeidbare Krankheit, den Impfstoff, die Impfung sowie über Impfreaktionen und mögliche Impfkomplikationen enthalten.

Before the vaccination is administered, we request the following additional information:
Vor Durchführung der Impfung wird zusätzlich um folgende Angaben gebeten:

1. Does the person to be vaccinated have an immunodeficiency (congenital, acquired or caused by medication)?
Besteht bei dem Impfling eine Immunmangelkrankheit (erworben, angeboren, durch Medikamente bedingt)?

□ Yes *(Ja)* □ No *(Nein)*

2. Does the patient have any known allergies?
Ist bei dem Patienten eine Allergie bekannt?

□ Yes *(Ja)* □ No *(Nein)*

If yes, which ones?: ____
wenn ja, welche?

3. Did the person to be vaccinated receive immunoglobulin or a blood transfusion in the last 3 months?
Hat der Impfling in den vergangenen drei Monaten ein Immunglobulin erhalten oder wurde eine Bluttransfusion vorgenommen?

□ Yes *(Ja)* □ No *(Nein)*

4. For vaccinations of women of childbearing age: are you currently pregnant?
Bei Impfung von Frauen im gebärfähigen Alter: Besteht zurzeit eine Schwangerschaft?

□ Yes *(Ja)* □ No *(Nein)*

If you would like to know more about the vaccination against chickenpox, please ask the vaccinating physician.
Falls Sie noch mehr über die Schutzimpfung gegen Windpocken wissen wollen, fragen Sie den Impfarzt!

Please bring your vaccination record to your vaccination appointment.
Zum Impftermin bringen Sie bitte das Impfbuch mit!

Declaration of consent

Einverständniserklärung

to vaccinate the person listed below against chickenpox (varicella)

zur Durchführung der Schutzimpfung gegen Windpocken (Varizellen)

Name of person to be vaccinated: ____

Name des Impflings

Date of birth: ____

geb. am

I have read the information leaflet, and have received detailed information about the vaccination during the consultation with my physician.

Ich habe den Inhalt des Merkblatts zur Kenntnis genommen, und bin von meinem Arzt/meiner Ärztin im Gespräch ausführlich über die Impfung.

□ I have no further questions.
Ich habe keine weiteren Fragen.

□ I give my consent to the recommended vaccination against chickenpox
Ich willige in die vorgeschlagene Impfung gegen Windpocken ein.

□ I decline the vaccination. I have been informed about the possible disadvantages of declining to be vaccinated.
Ich lehne die Impfung ab. Über mögliche Nachteile der Ablehnung dieser Impfung wurde ich informiert.

Notes: *Vermerke*____

Place and date: *Ort, Datum*____

________________________________	________________________________
Signature of person to be vaccinated or his/her legal guardian<br>*Unterschrift des Impflings bzw. des Sorgeberechtigten*	Doctor's signature<br>*Unterschrift des Arztes/der Ärztin*

about vaccinations against tetanus (lockjaw), diphtheria, pertussis (whooping cough), and polio (poliomyelitis)

If the booster vaccinations against tetanus, diphtheria, whooping cough and polio are due at the same time, they can be administered starting at the age of 3 or 4 years (review current usage information) as a quadruple vaccine with a combination vaccine (Tdap-IPV). It contains less diphtheria and pertussis components than the combination vaccines used for primary immunization of infants and young children.

Lockjaw (tetanus) is a life-threatening infectious disease. The bacterium is found mainly in dirt and road dust and enters the skin through cuts, cracks, abrasions, or bites, and even through the most minor sites of injury, and then proliferates in the body and forms the disease-causing tetanus poison. Muscle cramps are characteristic of the very serious disease symptoms and can lead to attacks of suffocation when the respiratory muscles are affected. Despite modern treatment methods, over 10 to 20 percent of patients with tetanus die.

Diphtheria is a life-threatening infectious disease caused by the poison (toxin) of the diphtheria bacterium, which is transmitted mainly by airborne droplet infection. Before vaccinations were introduced, many children died from the infection that presented as throat, larynx, nose, and wound diphtheria, or they suffered damage to their heart muscle. The high participation in vaccinations has eliminated diphtheria, apart from few isolated cases, in Germany. However, since it continues to occur globally - including in some Eastern European countries - the risk of re-introduction and re-infection when travelling can occur at any time.

Whooping cough (pertussis) usually begins as a cold. With primary infections, severe spells of coughing occur after 1-2 weeks that can persist for several weeks or months. Often the paroxysmal attacks of coughing lead to shortness of breath and vomiting. During the first 6 months of life, the characteristic coughing may be absent; instead, respiratory failure is a threat. Whooping cough is dangerous because of serious complications such as lung and middle ear inflammation and permanent brain damage; event today, fatal courses of the disease occur. Particularly at risk are children with heart and lung disease, and infants. Over the past years, pertussis disease has increasingly been observed in adolescents and adults; it is possible to become ill with whooping cough several times. The course is generally milder and less typical than in childhood. This often leads to misdiagnosis of the cause. The most common symptom is a long-lasting, persistent cough that usually occurs at night. Unrecognized pertussis disease can lead an infant or small child to become infected.

Polio (poliomyelitis) is a viral disease that can lead to paralysis of the arms and legs, but also to respiratory paralysis and thus, suffocation. Most patients with paralysis suffer long-term damage. Since 1998, polio vaccinations are no longer performed with an oral vaccine but with a vaccine of killed polio virus (inactivated vaccine). Polio is no longer found in Europe. The disease is still common in some African and Asian countries (e.g., Afghanistan and Pakistan). The pathogens introduced by travelers from these regions can spread rapidly and lead to reintroduction of the disease when the population is not adequately vaccinated. Polio vaccinations must therefore be performed consistently, even today.

Vaccine

The vaccine against tetanus, diphtheria, pertussis and polio (Tdap-IPV) contains as essential components detoxified toxins (toxoids) of the tetanus and diphtheria pathogens, parts of the killed pertussis bacteria (acellular pertussis vaccine) and inactivated polio viruses that build immunity against the vaccinee. The vaccine is tolerated well because the amount of diphtheria and pertussis antigens is reduced. The vaccine is injected into the muscle. Your physician can provide you with information regarding the start and length of vaccine protection.

Who should be vaccinated?

The combination vaccine Tdap-IPV is suitable for vaccination starting from the age of 3 or 4 years (depending on the manufacturer). It is especially recommended for the booster immunization scheduled in the immunization passport for 9 to 17-year-olds as well as for additional necessary booster vaccinations in adulthood (once for all adults with the next required Td vaccination, women of childbearing age, caregivers of infants and infants living in the household, and/or before the birth of a child). Personnel of health services and community facilities should be vaccinated against pertussis in addition to tetanus, diphtheria and poliomyelitis. For a booster injection, 1 injection suffices. The combination vaccination can be given simultaneously with other vaccines; time intervals with other vaccinations do not have to be observed. If an indication for a whooping cough vaccination exists, it can be performed with a Tdap-IPV vaccine, even if the last Td vaccination was only 4 weeks before.

Who should not be vaccinated?

Anyone suffering from acute illness with a fever (above 38.5°C) that requires treatment should not be vaccinated. If a hypersensitivity to any vaccine component exists or if disease symptoms had occurred after a previous vaccination against tetanus, diphtheria, whooping cough, and/or poliomyelitis (singly or in combination with vaccinations against other diseases) the vaccinating doctor will consult with you about the possibility of further vaccinations. In pregnant women, the benefits and risks of Tdap-IPV vaccination must be carefully weighed. Vaccination can be performed during the period of breast-feeding.

Behavior after vaccination

The vaccinated person does not require any special attention; however, unusual physical stress should be avoided within 3 days of vaccination. The doctor should be informed prior to vaccination if children are prone to cardiovascular reactions or have known immediate allergic reactions.

Possible local and systemic reactions following vaccination

After vaccination, redness or painful swelling may frequently occur at the injection site in addition to the desired immunity and corresponding disease protection. This is the body's normal reaction to the vaccine and usually occurs within 1-3 days, and seldom persists longer. In rare cases, the vaccine reaction is delayed by up to 14 days. Also within 1-3 days after vaccination (rarely persisting longer) general symptoms such as headache, chills, nausea, diarrhea, and joint pain may occur. In less than 5 percent of cases, the temperature may increase slightly to moderately; very rarely to 39.9°C and above. The above-mentioned reactions are generally temporary and quickly subside without long-term effects.

Are there possible complications from the vaccination?
Vaccination complications, beyond the normal extent of a vaccine reaction, are very rare consequences that have a significant negative impact on the vaccinee's health. In rare cases, vaccination against whooping cough and polio (Tdap-IPV) can lead to a hypersensitivity reaction of the skin or the respiratory tract; isolated cases of immediate allergic reactions (anaphylactic shock) have also been reported. As with administration of other vaccines containing tetanus and diphtheria, generally temporary nervous system have been reported in isolated cases for the Tdap-IPV vaccine (usually of the peripheral nervous system, e.g., mono- and polyneuritis, neuropathy).

Consultation with the vaccinating doctor about possible side effects
In addition to this leaflet, your doctor is available to meet for a consultation.

In the event that, following vaccination, symptoms should occur that are beyond the above-mentioned temporary local and general reactions, your immunizing doctor will of course also be at your disposal for advice.

You can reach the vaccinating doctor at:

Disclaimer
Translation of the original information leaflet (Status: 11/2014) with kind permission of the German Green Cross Inc. (Deutsches Grünes Kreuz e.V.), on behalf of the Robert Koch Institute. The German text is authoritative, and no liability is assumed for any translation errors or the currency of this translation in case of subsequent revisions of the German original.

Information

Combination vaccination against measles, mumps and rubella

Measles, mumps and rubella are widespread illnesses caused by viral infections (measles virus, mumps virus and rubella virus), and, in the majority of cases, occur mostly in children but in adults, too. Since the vaccines to protect against measles, mumps and rubella are administered in one combined injection (so-called MMR vaccination), these illnesses and the vaccination are discussed here together.

There are no drug treatments to cure measles, mumps or rubella. Only the timely, complete and correct administration of the MMR vaccination can protect you from these illnesses.

Measles is an infection that is easily transmitted from person to person and frequently causes serious illness. Symptoms include high fever, cough, conjunctivitis and a typical skin rash (exanthema). The illness lasts for about 2 weeks. Frequent complications are pneumonia and middle ear infections. Approximately one in 1,000 to 2,000 measles patients will develop inflammation of the brain (encephalitis), and 30 percent of these cases are either fatal or can lead to permanent mental and physical impairment. Adolescents and adults are at particular risk from measles and more frequently experience complications.

Mumps is a viral infection involving fever, headaches and swollen salivary glands ("mumps"). About every 10th mumps patient develops meningitis, and occasionally encephalitis. A rare, but typical complication is hearing loss. Every 4th adolescent or adult male with mumps will experience swollen and inflamed testicles, which in rare cases can also lead to infertility.

Rubella is usually a mild viral infection involving fever, skin rash (exanthema) and swollen lymph nodes. It often runs its course without symptoms, but these patients can still infect those around them. If rubella occurs during pregnancy, the infection can be passed on to the unborn child, causing abnormalities of the eye and ear as well as heart and brain.

Vaccine

The Measles Mumps Rubella vaccine consists of weakened but live viruses, which then reproduce in the body. The vaccine is injected into a muscle (intra-muscularly) or under the skin (subcutaneously). Your doctor can tell you about the start and duration of the vaccine protection. According to the state of knowledge today, booster shots are not required after 2 MMR vaccinations.

Who should be vaccinated, and when?

The MMR combination vaccine is recommended for all boys and girls aged between 11 and 14 months. In order to achieve a safe level of immunity, all children aged between 15 and 23 months are vaccinated again. However, it is also possible to vaccinate again just four weeks after the first vaccination.

If a child is to be placed in a day-care facility, then the first MMR vaccination can also be administered earlier – however at 9 months at the earliest. If the first vaccination was administered before the age of 11 months, then it is important to give the 2nd vaccination just after the 1st birthday.

The German Standing Committee for Vaccination *(STIKO)* points out that vaccinations that have not been carried out at the recommended times should be caught up later, up to the age of 18 years.

Also, all adults born after 1970 who have been vaccinated in childhood against measles only once or not at all, or whose vaccination status is unknown, should receive a one-off vaccination, preferably with MMR vaccine. Furthermore, all those employed in health services, and working in community services as well as those in contact with persons with a weakened immune system should be vaccinated if they are not already protected against measles, mumps and rubella.

After contact with a person with measles, mumps or rubella, unvaccinated persons older than 9 months, or those who have been vaccinated only once or not at all, or whose vaccination status is unclear, should receive a one-off MMR vaccination, if possible within 3 days after contact.

Please note: for children and adolescents it should be checked whether they have already received 2 MMR vaccinations. The Measles Mumps Rubella vaccination poses no risk to people who are already immune to one or more of these illnesses. There is no upper age limit for the MMR vaccination.

If there is an outbreak of measles, all adults born after 1970 with an unknown vaccination status, who have not been vaccinated, or who have only been vaccinated once in their childhood should receive a one-off vaccination, preferably with MMR vaccine.

Who should not be vaccinated?

Individuals suffering from an acute illness with more than 38.5°C fever should be vaccinated at a later time. In general, persons with an immunodeficiency, be it congenital, acquired or caused by medication, should not be vaccinated; vaccination can be considered when the benefit outweighs the risk (e.g., with asymptomatic HIV-infection). If symptoms of diseases appeared after a first MMR vaccination, or if there is severe hypersensitivity against components in the vaccine, your immunizing doctor will advise you about a possible procedure. Persons with an allergy to chicken eggwhite (anaphylactic reaction after eating eggwhite) can usually be vaccinated, but the vaccination should be performed with appropriate precautionary measures in place.

The MMR vaccination should not be administered to persons who have received immunoglobulin within the past 3 months or who have had a blood transfusion during the same period (antibodies present in the donated blood may render the vaccination ineffective). The MMR vaccination should not be administered during pregnancy as there is a theoretical risk to the unborn child from the vaccine virus. For the same reason, pregnancy should be avoided for up to 1 month after vaccination. However, a vaccination administered accidentally during a pregnancy is not a reason for abortion.

After the vaccination

The vaccinated person (vaccinee) does not need to take special care, but higher than usual physical exertion should be avoided for 1 to 2 weeks after vaccination. The vaccinating physician should be informed before vaccination of any tendencies towards circulatory problems or sudden onset allergic reactions.

The weakened vaccine viruses cannot be transmitted from person to person, i.e. vaccinating a child poses no risk to pregnant women in close proximity.

Possible localised and general reactions to the vaccination

As well as providing the intended immunity, to protect from the illness, for about 5 percent of the vaccinated persons the vaccination can result in some discomfort, including soreness, swelling and redness at the injection site. This is the body’s normal way of dealing with the vaccine and occurs within 1 to 3 days after the vaccination, rarely lasting very long. Occasionally, nearby lymph nodes can become swollen. General symptoms like headache, weariness, feeling unwell or gastrointestinal complaints can occur frequently. Between 5 and 15 percent of vaccinated persons experience a slightly or moderately elevated temperature.

One to two weeks after the vaccination, approximately 2 percent of vaccinated persons show slight signs of a measles, mumps or rubella infection. This is a non-infectious, so-called vaccination illness. Fever, for example, can occur together with a mild, measles-like rash. Sometimes the parotid gland becomes slightly swollen. Temporary joint pain has been observed in some adolescents and adults, but very rarely in children. Very rarely the testicles can become slightly swollen or the pancreas may react mildly and temporarily (through an enzyme increase).

As a rule, these are all of a temporary nature, and subside quickly and without lasting effects.

What about vaccination complications?

Complications are unintended effects beyond usual vaccine reactions and affect the vaccinated person's health significantly. If an infant or young toddler reacts to the MMR vaccination with fever, there is a slight possibility of developing a febrile seizure. These generally have no lasting effect. Allergic reactions are very rare. They are mostly caused by the secondary components of the vaccine, such as gelatine or antibiotics. Hardly any cases of anaphylactic shock have been reported. There have also only been isolated cases of skin bleeding after vaccination as a result of a reduced platelet count. They usually subside quickly with no lasting effects. Serious developments occur only in isolated cases. On very rare occasions, longer periods of inflammation of the joints have been observed in adolescents and adults. Concerning locally licenced mumps vaccines made from the 'Jeryl Lynn' strain, sporadic cases of meningitis seem possible, but have so far not been virologically confirmed. A causative connection with other disorders of the neurological system that appeared at the same time as the MMR vaccination, which was reported in rare cases, is also questionable.

Please note: allergies to chicken egg white is no reason to avoid MMR vaccination as measles and mumps vaccine viruses are not grown in eggs, but using embryonic chicken cells. This means that chicken egg white (ovalbumin) is present, at most, as small and almost undetectable traces, which are therefore not harmful

Physician's advice on side effects

In addition to this information leaflet your doctor is offering a personal consultation.

If, after a vaccination, symptoms occur that go beyond the rapidly subsiding local and general reactions described above, the vaccinating doctor will also be available to advise you.

You can contact the vaccinating doctor here:

Disclaimer

Translation of the original information leaflet (Status: 09/2014) with kind permission of the German Green Cross Inc., on behalf of the Robert Koch Institute. The German text is authoritative, and no liability is assumed for any translation errors or the currency of this translation in case of subsequent revisions of the German original.

Information

Vaccination against Chickenpox (varicella)

Chickenpox is a highly contagious illness caused by the varicella zoster virus, and is widespread throughout the world. It is spread by direct body contact or via the air (airborne droplet infection). About 2 weeks after becoming infected, the typical rash begins to appear, mostly accompanied by fever. Small, isolated red patches quickly turn into lentil-sized blisters filled with fluid. These become cloudy, dry out and form a scab after a few days. Typically, the spots are intensely itchy.
Normally, someone with chickenpox is most infectious 2 days before the rash appears until 5 days after the last fresh blisters have formed. After the rash has healed, the virus remains dormant in the nerve cells (ganglia) in the bone marrow and brain. It can become reactivated and cause shingles (zoster), mostly in much older patients and in persons with weakened immune systems.

Serious complications during chickenpox, for example bacterial superinfections of the skin, inflammations of the brain or lungs are rare in healthy children. In contrast, adolescents and adults have a significantly higher risk of complications. Particularly
at risk are all unprotected patients whose immune system is weakened, be it due to a congenital or an acquired dysfunction, or one caused by medication. For children with leukemia, chickenpox can even be fatal.

Chickenpox is also dangerous for pregnant women if they have neither had chickenpox nor had been vaccinated against it. Chickenpox infections during the first 20 weeks of pregnancy can cause serious birth defects in the unborn child. If an unprotected mother becomes ill with chickenpox around the time of birth (5 days before and up to 2 days after giving birth), a chickenpox infection contracted by the newborn can be life-threatening. Even for premature babies of protected mothers, an infection during the first six weeks of life is dangerous.
When newborn babies and patients with a weakened immune system catch chickenpox, antiviral drugs can be administered to avoid serious complications. However, only timely vaccination provides real protection against all types of disease progression caused by varicella infections.

Vaccine

The vaccine consists of live but weakened varicella zoster viruses, which then reproduce in the body. The vaccine is injected under the skin (subcutaneous injection). The vaccination against chicken pox can be given from the age of (9 to) 12 months.
Your doctor can tell you about the start of the vaccine protection. According to the state of knowledge today, booster shots are not required after 2 varicella vaccinations.

Who should be vaccinated?

Within the framework of the vaccination schedule, the chickenpox vaccination is recommended for all children and should normally be given between 11 and 14 months of age. In order to ensure a safe level of immunity, children are vaccinated again between 15 and 23 months of age. However, the second dose can be given as early as (4 to) 6 weeks after the first. Children who have only been vaccinated once should receive a catch-up dose. The vaccination can be performed either along with the vaccinations against measles, mumps and rubella (MMR vaccination), or 4 weeks later at the earliest.

If the following individuals have not gone through chickenpox yet, and have not been vaccinated either, then the 2-dose vaccination is generally recommended for:

- Children and adolescents up to their 18th birthday,
- Women intending to have a child,
- (Newly employed) staff in childcare facilities for pre-school children (e.g.

kindergarten),

- Medical staff, especially in birthing units, pediatrics, oncology, intensive care and those caring for patients with immune deficiencies.

In addition, the vaccination is recommended for all vulnerable individuals for whom an infection would be particularly dangerous, or who could spread chickenpox to people at risk. These are primarily tumor patients, patients with serious atopic dermatitis and those scheduled for surgery or treatments that weaken the immune system (immunosuppressive therapy), such as prior to organ transplants, or those with severe kidney diseases. Moreover, it is imperative for persons who are in close contact with these patients (family members, or careers), as well as the medical staff, to be vaccinated.

Under certain circumstances, the vaccination can still provide protection even if administered a few days after infection. In these cases, please consult a doctor immediately.

Who should not be vaccinated?

A person suffering from an acute illness with more than 38.5°C fever and requiring treatment should not be vaccinated. Persons who are allergic to components in the vaccine must not be vaccinated. In general, individuals with a weakened immune system are also not vaccinated. Exceptions are possible and necessary under certain circumstances. The varicella vaccination should not be given during a pregnancy, since a theoretical risk for the child by the vaccination virus exists in the womb. For the same reason, pregnancy should be avoided for a period of at least 1 month after vaccination. However, a vaccination administered accidentally during a pregnancy is not a reason for abortion.
So as not to jeopardize the success of the vaccination, it is not administered within at least three months after receiving either immunoglobulin or a blood transfusion.

In all these cases, the vaccinating doctor will be happy to advise you on how you can best protect yourself from varicella infection.

After the vaccination

The vaccinated person (vaccinee) does not need to take special care, but higher than usual physical exertion should be avoided for 1 to 2 weeks after vaccination. The vaccinating physician should be informed before vaccination of any tendencies towards circulatory problems or sudden onset allergic reactions.

In extremely rare cases, vaccine viruses can be transmitted to susceptible contacts. This is why persons with a severely weakened immune system and non-immune pregnant women should avoid contact with vaccinated persons who have reacted to the vaccination with isolated chickenpox blisters. They should avoid such contact until 5 days after the appearance of the last fresh blisters.

Since there is a theoretical risk of Reye syndrome, no salicylates (e.g. aspirin) should be taken for a period of 6 weeks after the vaccination.

Possible localised and general reactions to the vaccination

As well as achieving the intended immunity to protect from the illness, about 20 percent of vaccinated persons, particularly adults after the 2nd vaccine dose, will temporarily experience some redness or painful swelling at the injection site. This is the body's normal way of dealing with the vaccine. Approximately every 10th vaccinated person (vaccine) will experience a slight to moderate temperature. In 1 to 3 percent, a very mild form of chickenpox (vaccination illness) occurs within 1 to 4 weeks after vaccination, including isolated blisters and fever. In patients with a weakened immune system, these symptoms are more severe and occur more frequently. This means that, as a rule, these individuals should not receive this vaccine. It should, however, be given to children with leukemia in remission (a period of time without symptoms).

What about vaccination complications?

Complications are unintended effects beyond usual vaccine reactions and affect the vaccinated person's health significantly. Allergic reactions after varicella vaccination are very rare. Vaccinations for chickenpox must not be given if there is a framycetin/neomycin intolerance. Isolated cases of sudden onset of allergic reactions including anaphylactic shock, shingles and pneumonia in healthy individuals and people with a weakened immune system have been reported in the literature. Similarly, only isolated cases of transmission from a vaccinated person with vaccine illness (skin rash) to a contact person – mostly with a weakened immune system – have been reported as well as a transient decline in platelets. A causative connection with other disorders of the neurological system that appeared at the same time as the varicella vaccination, which was reported in rare cases, is also questionable.

Physician's advice on possible side effects

In addition to this information leaflet, your doctor is offering a personal consultation.

If, after a vaccination, symptoms occur that go beyond the rapidly subsiding localized and general reactions described above, the vaccinating doctor will also be available to advise you.

You can contact the vaccinating doctor here:

Disclaimer

Translation of the original information leaflet (Status: 09/2014) with kind permission of the German Green Cross Inc., on behalf of the Robert Koch Institute. The German text is authoritative, and no liability is assumed for any translation errors or the currency of this translation in case of subsequent revisions of the German original.
